# Lessons from a century of apical dominance research

**DOI:** 10.1093/jxb/erad137

**Published:** 2023-04-20

**Authors:** Christine A Beveridge, Catherine Rameau, Akila Wijerathna-Yapa

**Affiliations:** ARC Centre of Excellence for Plant Success in Nature and Agriculture, St Lucia, QLD 4072, Australia; School of Biological Sciences, The University of Queensland, St Lucia, QLD 4072, Australia; Université Paris-Saclay, INRAE, AgroParisTech, Institut Jean-Pierre Bourgin (IJPB), 78000, Versailles, France; ARC Centre of Excellence for Plant Success in Nature and Agriculture, St Lucia, QLD 4072, Australia; School of Biological Sciences, The University of Queensland, St Lucia, QLD 4072, Australia; MPI of Molecular Plant Physiology, Germany

**Keywords:** Apical dominance, auxin, axillary bud, cytokinins, genetics, physiology, shoot branching, strigolactones, sugars, tillering

## Abstract

The process of apical dominance by which the apical bud/shoot tip of the plant inhibits the outgrowth of axillary buds located below has been studied for more than a century. Different approaches were used over time, with first the physiology era, the genetic era, and then the multidisciplinary era. During the physiology era, auxin was thought of as the master regulator of apical dominance acting indirectly to inhibit bud outgrowth via unknown secondary messenger(s). Potential candidates were cytokinin (CK) and abscisic acid (ABA). The genetic era with the screening of shoot branching mutants in different species revealed the existence of a novel carotenoid-derived branching inhibitor and led to the significant discovery of strigolactones (SLs) as a novel class of plant hormones. The re-discovery of the major role of sugars in apical dominance emerged from modern physiology experiments and involves ongoing work with genetic material affected in sugar signalling. As crops and natural selection rely on the emergent properties of networks such as this branching network, future work should explore the whole network, the details of which are critical but not individually sufficient to solve the ‘wicked problems’ of sustainable food supply and climate change.

## Teaser

The importance of multidisciplinary and multispecies approaches is illustrated through the review of apical dominance and shoot branching research over the last century.

## Introduction

**Fig. 1.**
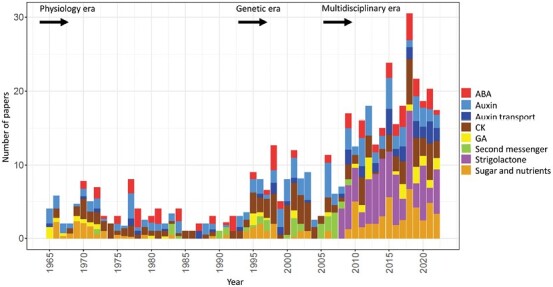
Branching-focused research output over the years. The Clarivate Analytics Web of Science was used to search outputs from 1 January 1965 to 20 October 2022 with ‘shoot branching’ OR ‘apical dominance’ OR ‘axillary bud’ OR ‘bud outgrowth’ OR ‘bud dormancy’ in Topic (which searches the title, abstract, author keywords, and keywords plus). Search results were refined for document types of article and early access, and Web of Science categories of Plant Sciences, Horticulture, Agronomy, Biochemistry and Molecular Biology, and Multidisciplinary Sciences, producing 2436 articles. Each publicly available article was screened manually and categorized. Papers were selected across eight branching hypothesis bins including abscisic acid (ABA), auxin, auxin transport, cytokinin (CK), gibberellin (GA), secondary messenger, strigolactone, and sugar/nutrients. Papers were excluded that either did not focus on the branching topic or were mostly descriptive rather than hypothesis testing, leading to 483 hypothesis-testing articles. Articles covering multiple bins were plotted with a contributed fraction to each associated bin.

Of course, science has changed dramatically over the last 100 years. The rate of discovery and the amount of technical change and innovation are truly impressive (Fig. 1). What can we learn from the experiences of that change; how has it impacted our current status in the field of science, and how should we move forward? These are big questions to which a response from only one perspective can be offered here, and that is from the point of view of apical dominance/shoot branching research.

In the 1920s, physiology was the name of the game, and it included biochemistry, anatomical, and developmental research. This was followed by the genetic era (Fig. 1). While mutants may initially have been regarded by holistic physiologists as too specific or not representative of reality, mutants identified by phenotype were soon regarded as the bread and butter of the fundamental research approach. As DNA technologies became available, a whole PhD in the 1980s could be spent on cloning a single gene underlying a mutant phenotype (forward genetics). Soon the production of transgenic plants became possible, and the Arabidopsis genomics era emerged together with molecular biology and reverse genetics. The advent of the powerful technique of gene editing came much later. At the time of the genetic era, physiology itself, which is naturally a holistic study, was somewhat overshadowed by a reductionist approach of one gene, protein, or molecular process at a time. Following this, transcriptomics, genomics, and phenomics generated immense datasets in the omics era. However, the promise of the ‘virtual plant’ and the understanding of the function of all genes in a plant genome did not in itself guarantee that a holistic understanding of plants was attainable. The sheer rate and volume of discovery encouraged research focused on molecular mechanisms and details rather than on interactions among all plant parts. However, current multipronged and multidisciplinary approaches enable a bigger picture to be facilitated. In many ways, this bigger picture approach re-visits the theories and approaches of the physiology era. It is from within this context of different eras of research that we review the topic of apical dominance and shoot branching, with a focus on herbaceous plants.

## Apical dominance (physiology era)

Apical dominance occurs when a shoot tip suppresses axillary bud outgrowth and restricts the number of branches. The experiments performed during the ‘physiology era’ focused on the discovery of the physiological mechanisms of apical dominance and frequently involved studies of herbaceous plants with strong apical dominance, such as annual legumes (e.g. pea, bean, and soybean). However, apical dominance is only one aspect of the processes controlling shoot branching, particularly in tree species. Box 1 describes other aspects of bud inhibition that are not reviewed herein. We acknowledge that researchers often use the term ‘dormant’ in annual plants to describe buds that are strongly inhibited even though the buds are highly metabolically active and slow growing (see quiescent, Box 1) (Stafstrom and Sussex, 1988).

**Box 1. Definitions**

Clear definitions of the different types of bud dormancy observed in herbaceous and perennial plants have been described (Lang *et al.*, 1985, 1987; Cooke *et al.*, 2012; Considine and Considine, 2016; Yamane *et al.*, 2021). This review focuses on branching in annual plants. Due to the common use of the word dormant to describe the slow growing, inhibited, and quiescent bud of many annual plants, we continue to use the word dormant in this context.

**Apical dominance**: also termed paradormancy, is when growth of the lateral bud is inhibited by the growing apical bud/shoot.

**Bud release:** the initial phase of a bud response to commence growth, sometimes termed bud activation.

**Dormant bud**: is a context-dependent term used in woody plants to mean no growth, with little or no metabolic activity; and in annual plants, to mean no or very little growth and typically metabolically active.

**Correlative dominance**: when growth of the lateral bud is inhibited by other actively growing branches or organs (e.g. flowers and fruits).

**Ecodormancy**: when the bud is competent to resume growth but limiting environmental factors such as nutrients or water inhibit its outgrowth.

**Endodormancy**: induced by environmental factors (e.g. low temperatures or short day length) or endogenous factors and when growth can resume only after a certain period of low temperatures or others environmental cues.

**Quiescent bud**: bud with meristem where cell division is arrested (and can resume growth without delay).

**Subsequent growth**: is the phase between bud release and sustained growth, sometimes termed post-activation.

**Sustained growth**: where the actively growing axillary shoot is no longer clearly subject to control by apical dominance (but may be affected by correlative inhibition).

Humans have long taken advantage of shoot tip pruning to promote a bushy phenotype. This plasticity of plant development captured the interest of scientists long before genetic control of development was described. Removal of the shoot tip, or pruning, typically enables the growth of axillary buds. The nutrient diversion theory (reviewed in Phillips, 1975) emerged because it was thought that the removed growing parts enabled more resources to flow to axillary buds.

Early alternatives to the nutrient diversion theory suggested possibilities that were nevertheless related to nutrients. For example, inhibited buds may lose the ability to absorb nutrients or may have poor connectivity to the stem (reviewed in Phillips, 1975). The role of water in its capacity to promote turgor-driven growth was also investigated (McIntyre, 1987). Snow (1925) was one of the most cited early proponents of an alternative to the nutrient diversion theory and defined experiments that supported the hypothesis of an inhibitory substance (or hormone) that arises in the shoot tip. Snow (1929) also showed that an inhibitory signal could be transmitted acropetally through a section of dead stem. After the early discoveries of plant hormones, the nutrient theory was connected to hormones in relation to hormone-directed assimilate transport (Phillips, 1975; Cline, 1997).

Went (1926) developed a cut tip bending bioassay for a hormone produced in the shoot tip, which enabled the discovery of ‘the growth hormone’ auxin (Thimann and Skoog, 1933; Skoog and Thimann, 1934). Auxin produced in the young and growing leaves of the shoot tip was suggested to inhibit axillary buds. The first few experiments with auxin treatments showed that the inhibition of buds by exogenous auxin was not associated with the growth of other shoot parts (Thimann and Skoog, 1933, 1934). This auxin inhibition theory was therefore suggested as a replacement for the nutrient diversion theory (Phillips, 1975), although the latter retained some relevance throughout the years, particularly with reference to the general requirement for photoassimilates and inorganic nutrients for growth (Fig. 1).

Clues that something in addition to auxin may have been at play in inhibiting bud outgrowth included two important observations. First, the amount of auxin required to inhibit branching after decapitation was very high (Thimann, 1937 used auxin at 4% of the medium supplied; reviewed by Cline, 1997). Second, as mentioned above, some kind of inhibitory signal can move through the xylem (Snow, 1929). Evidence began to emerge that the endogenous indole-3-acetic acid (IAA) form of auxin could not move upwards into buds, and yet auxin could exert an acropetal effect. For example, the classic two-shoot ‘W’ experiment of Snow (1937) showed that an intact shoot can inhibit branching in a decapitated shoot of an adjacent plant attached via a graft union. This study and several referred to therein strongly supported the indirect theory/secondary messenger theory whereby auxin exported from shoot tips affects the level or transport of another signal(s).

Partly to account for indirect auxin action and partly due to the evidence of auxin regulation of vascular development (Sachs, 1969, 1981), the concept of auxin transport and canalization emerged as an important driver of bud inhibition. The well-developed vasculature of axillary buds in certain plants wherein branching could be activated by shoot tip removal (decapitation) made this hypothesis difficult to establish unequivocally. One of the strong aspects of auxin transport theory in the relatively early literature was the correlation of vigorous growth and auxin transport in one shoot with reduced auxin transport in a subordinate shoot (Morris, 1977; Li and Bangerth, 1999). Interestingly, in his major review, Cline (1997) makes a strong distinction between apical dominance and correlative dominance (Box 1), a point of difference that remains relevant. The second messenger hypothesis was, therefore, not mutually exclusive of the nutrient diversion and auxin transport theories, which, in turn, were applicable to both forms of dominance.

The discovery of cytokinins (CKs) in the 1950s by Miller and colleagues (Miller *et al.*, 1955, 1956) led to rapid advances in understanding shoot branching, as exogenous CK could promote branching even in the presence of a growing shoot tip (Sachs and Thimann, 1964). This led to the hypothesis that auxin enhances CK levels which can move acropetally in the shoot and into buds (Bangerth, 1994; Li *et al.*, 1995; Shimizu-Sato *et al.*, 2009). The timing of changes in endogenous CK and auxin levels was reported to correlate with the timing of bud outgrowth in several experiments (Turnbull *et al.*, 1997; Tanaka *et al.*, 2006; Shimizu-Sato *et al.*, 2009). This correlation was not strictly tested as, in each case, the experimental design involved a short distance (and therefore timing) from induced changes in auxin content and the measured outgrowing buds. Furthermore, although many early reports discussed that CKs alone were often insufficient to promote the continued growth of axillary buds (Phillips, 1975), this was rarely mentioned once studies showed that a single dose of CK could indeed be effective (Pillay and Railton, 1983).

With regards to the hypothesis of a second messenger acting downstream of auxin, auxin regulation of CKs nicely explained the indirect action of auxin. However, other hormones such as abscisic acid (ABA) and gibberellins (GAs) remained as candidates for second messengers or additional signals due to the evidence that their levels were often correlated with bud outgrowth (Fig. 1). For example, Tucker and Mansfield (1971) showed that far-red light treatment induced high endogenous ABA levels in tomato plants and exogenous ABA treatment caused suppression of lateral bud development even in decapitated plants, and thus proposed ABA as a second messenger for auxin (Tucker and Mansfield, 1971; Tucker, 1977). Similarly, studies have repeatedly shown that GA is effective at promoting bud elongation after an initial period of growth stimulated by decapitation or CKs (reviewed in Ali and Fletcher, 1970; Phillips, 1975).

**Fig. 2.**
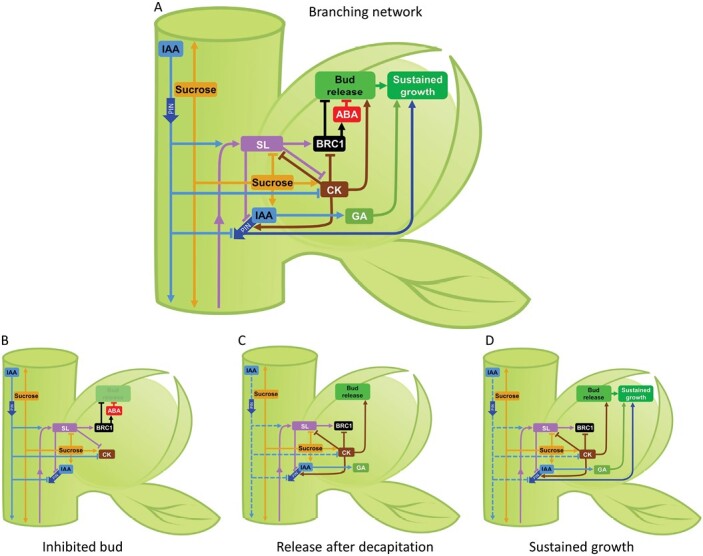
Simplified model of the hormonal control of branching showing (A) the whole branching network and (B and C) stages involved in producing a branch once an axillary bud is formed using the example of branching after decapitation; (B) an inhibited bud, (C) initial bud release after decapitation, and (D) the sustained growth stage. Apically derived auxin (IAA) stimulates SL biosynthesis, reduces CK levels and represses export of IAA from the bud. SL up-regulates BRC1 expression. SL also represses IAA export from the bud by acting on PIN polarity at the plasma membrane. SL may also act by a BRC1-independent pathway(s). CK has the opposite role to SL, and there is some feedback between SL and CKs. BRC1 acts to inhibit bud release and may act partly through ABA, although the role of ABA is unclear. Sucrose inhibits SL and promotes CK and IAA. Enhanced IAA levels and transport from the bud promote sustained growth, at least partly through enhancing GA. Buds are inhibited (B) primarily due to high SL levels and a poor sugar supply. Decapitation (C) induces bud release by reducing stem IAA, and enhancing sucrose supply, reducing SL, and enhancing CK in the bud. Sustained growth (D) will follow even if stem IAA and sucrose are restored, provided that IAA-enhanced GA levels cause bud elongation and enhanced auxin transport continues from the bud. Hormone levels and signalling are not shown separately. Potential effects of SL that are independent of both BRC1 and IAA transport are not shown. The dashed lines for IAA in C and D are to represent nodes either close to, or at a distance from, the decapitation site; depending on their position, these nodes may, or may not, have depleted IAA content. Arrows indicate promotion and blunt ends indicate suppression.

Shoot branching can involve stages, as shown in Fig. 2. Buds transform through cycles of bud growth activation, repression, and reactivation, a phenomenon that held some attention during the physiology era (Stafstrom and Sussex, 1988; Stafstrom, 1993; Napoli *et al.*, 1998). Stafstrom and colleagues showed the presence of differentially abundant proteins at the different stages, which include dormant or very slow-growing buds, released buds, released buds undergoing suppression, and sustained growth (Stafstrom, 1993; Fig. 2). This phenomenon is discussed again later as it was re-explored recently in the context of the current understanding of shoot branching (Fig. 2; Barbier *et al.*, 2015a; Cao *et al.*, 2023).

## Mutants and molecular approaches (genetic era)

Since the discovery of the fundamental laws of inheritance in the garden pea by Mendel, several species have been used as genetic models, including pea (*Pisum sativum*), tomato (*Solanum lycopersicum*), petunia (*Petunia hybrida*), snapdragon (*Antirrhinum majus*), rice (*Oryza sativa*), maize (*Zea mays*), and Arabidopsis (*Arabidopsis thaliana*). Large collections of mutants based on chemical-, radiation-, and transposon-based mutagenesis were developed in the 1900s, and forward genetic screens were performed. These early studies led to major discoveries of mobile DNA elements and epigenetics (reviewed in Nannas and Dawe, 2015; Borrill, 2020). In the 1980s, many plant biologists decided to concentrate on one model plant, Arabidopsis, largely because of its small genome that allows easy gene cloning and the ability to perform saturated mutagenesis and transformation (e.g. Meyerowitz, 2001). Intensive research on Arabidopsis has led to major insights into fundamental processes of plant biology, particularly at the molecular level.

Studies on shoot branching from the 1990s reflect the evolution of plant science with its emphasis on the power of genetics and the value of using several model species with complementary assets for novel and key discoveries. Combining the genetic approach (mutants) with ‘classical’ physiology (grafting and hormone quantifications) and gene cloning in multiple species led to the identification of the novel branching inhibitor (Gomez-Roldan *et al.*, 2008; Umehara *et al.*, 2008) and the first model for the genetic control of shoot branching (Beveridge, 2000).

Grafting studies and hormone quantifications with the *ramosus* (*rms*) mutants in garden pea led to the hypothesis of the existence of a novel branching inhibitor that was not auxin or CK (Beveridge *et al.*, 1997b). The first genetic branching model was proposed with pea mutants categorized as branching inhibitor response mutants and branching inhibitor biosynthesis mutants (reviewed in Beveridge, 2000). An additional shoot to root signal, coined the RMS2 feedback signal, was also described as involved in the regulation of xylem CK content, the novel branching inhibitor, and auxin itself (Beveridge *et al.*, 1997a, b; Beveridge, 2000; Foo *et al.*, 2001; Morris *et al.*, 2001). In parallel, several other papers were published between 1994 and 2005 (Fig. 1) or soon after, in which branching mutants were characterized in Arabidopsis (Stirnberg *et al.*, 2002; Sorefan *et al.*, 2003; Booker *et al.*, 2004, 2005), rice (Ishikawa *et al.*, 2005; Zou *et al.*, 2005, 2006; Arite *et al.*, 2007), and petunia (Snowden *et al.*, 2005; Simons *et al.*, 2007; Drummond *et al.*, 2009), and the corresponding genes named. The first branching genes to be cloned were the Arabidopsis *MORE AXILLARY BRANCHING* (*MAX*) genes (*MAX2*, Stirnberg *et al.*, 2002; *MAX4*, Sorefan *et al.*, 2003; *MAX3*, Booker *et al.*, 2004, and *MAX1*, Booker *et al.*, 2005). Homologues of the *MAX* genes were shown to be altered in the rice, pea, and petunia branching mutants, and additional new genes were identified in rice (Arite *et al.*, 2009; Lin *et al.*, 2009) and later reported in other species. These studies in eudicot and monocot plants indicated that the branching inhibitor pathway was probably conserved in plants. The *RMS2* gene has recently been identified in pea (Ligerot *et al.*, 2017) and will be discussed later in this review.

## Discovery of strigolactones (SLs) and elucidation of the branching network (multidisciplinary era)

Functional analysis of the *MAX3* and *MAX4* Arabidopsis genes (Sorefan *et al.*, 2003; Booker *et al.*, 2004) involved in the SL branching inhibitor biosynthesis pathway revealed that these genes encoded CAROTENOID CLEAVAGE DIOXYGENASES (CCD7 and CCD8, respectively). At that time, it was known that other CCDs metabolized carotenoid-type compounds (similar to the ABA hormone pathway). This was a crucial step in discovering the structure of the elusive SL branching inhibitor. Another critical step was achieved by Matusova *et al.* (2005) in the very different field of research on parasitic plants. It was demonstrated that a family of compounds, SLs, found in root exudates of plants and well known for several decades as potent germination stimulants of certain parasitic weeds, were carotenoid derived. SLs exuded from plant roots are essential for parasitism to occur. During the same period, the same SL molecules were shown to stimulate the hyphal branching of arbuscular mycorrhizal (AM) fungi (Akiyama *et al.*, 2005; Besserer *et al.*, 2006), a process essential for nutrient uptake in ~80% of land plants. Therefore, the branching community was looking for the identity of a mysterious carotenoid-derived branching inhibitor. In contrast, the two other communities of researchers studying parasitic plants and AM symbiosis were looking for the genes (and for mutants) involved in the biosynthesis and signalling pathways of SL. The *CCD* gene family contains nine genes in Arabidopsis, five *9-NINE-CIS-EPOXYCAROTENOID DIOXYGENASE* (*NCED*) genes being involved in ABA biosynthesis, and four *CCD* genes (*CCD1*, *CCD4*, *CCD7*, and *CCD8*) (Auldridge *et al.*, 2006). As SL was shown to be carotenoid derived, the best candidate genes for being involved in the SL biosynthesis pathway were the other four *CCD* genes, including *CCD7* and *CCD8*, which were known to be involved in the biosynthesis of the novel branching inhibitor.

Crop species, garden pea and rice, were pivotal in the discovery of SL as the branching hormone. While endogenous canonical SLs could be readily found in rice and pea roots and exudates, this was not the case for Arabidopsis (Xie, 2016; Wang and Bouwmeester, 2018; Yoneyama *et al.*, 2018b). These crop species are able to establish AM symbioses, and although several species of Orobanche can parasitize Arabidopsis, the germination stimulants of Arabidopsis were unknown (Bouwmeester *et al.*, 2003). Therefore, two independent research groups working on rice and pea discovered that SL was a novel family of plant hormones (Gomez-Roldan *et al.*, 2008; Umehara *et al.*, 2008), with each group studying shoot branching in these different crop species and using associated knowledge of the role of SLs in parasitic weeds and mycorrhizal symbiosis.

Removal (mutants) and replacement (exogenous SL treatment) experiments involving shoot branching, parasitism, and mycorrhizal associations enabled SLs to be discovered as the novel branching hormone (Gomez-Roldan *et al.*, 2008; Umehara *et al.*, 2008). Furthermore, the findings matched the predictions of prior physiological and genetic studies with respect to branching inhibitor biosynthesis genes acting at sequential steps in the biosynthetic pathway of SLs and branching inhibitor response genes required for SL signalling (e.g. reviewed in Beveridge *et al.*, 2009; Beveridge and Kyozuka, 2010).

From 2008 onwards, a large number of papers on SL biosynthesis and signalling pathways were published (many not included in the branching-specific articles in Fig. 1), together with studies for deciphering how SL and other signals acted together (Figs 1, 2). One key question was to test whether SL could be the secondary messenger acting downstream of IAA (see below). Here, because numerous excellent reviews have been published on the SL biosynthesis and signalling pathways (Jia *et al.*, 2018; Yao *et al.*, 2018a; Machin *et al.*, 2020; Tal *et al.*, 2020; Temmerman *et al.*, 2022), after a brief summary we will highlight some of the current key questions concerning the mode of action of SL in shoot branching.

**Fig. 3.**
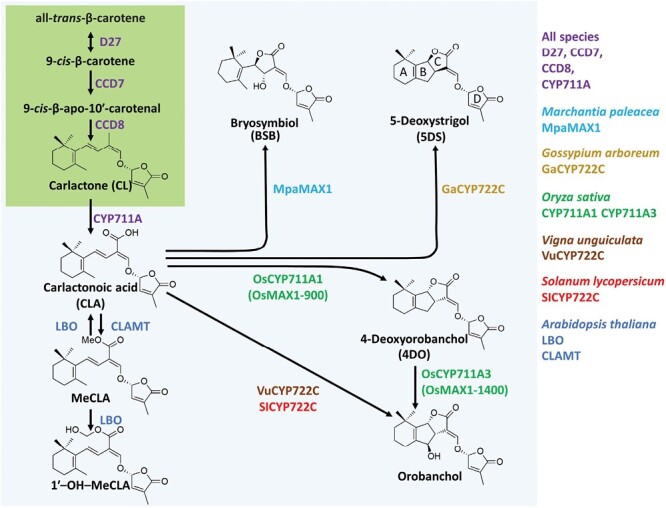
Simplified SL biosynthesis pathways in different species. The core pathways leading to CL are shown in a green box. CL is the precursor of both canonical and non-canonical SL after its conversion to CLA by the CYP711A/MAX1 subfamily. Canonical SLs have the 4 rings labeled A–D as shown in 5DS.

SL biosynthesis (Fig. 3) starts with the isomerization of all-*trans*-β-carotene into 9-*cis*-β-carotene catalysed by DWARF27 (D27) (Lin *et al.*, 2009), followed by cleavage and rearrangement reactions by CCD7 and CCD8, to give the biosynthetic intermediate carlactone (CL) (Alder *et al.*, 2012). In Arabidopsis and other species, the cytochrome P450 monooxygenase CYP711A/MAX1 and homologues catalysed the oxidation of CL to produce carlactonoic acid (CLA) (Abe *et al.*, 2014; Yoneyama *et al.*, 2018a). In other species, MAX1 homologues show functional diversity downstream of CLA. In rice, one of the five MAX1 homologues, Os900, catalysed the conversion of CL into 4-deoxy-orobanchol (4DO); and Os1400 catalysed the hydroxylation of 4DO to produce orobanchol (Zhang *et al.*, 2014). In cowpea and tomato, another CYP450, CYP722C, catalysed the direct conversion of CLA to orobanchol (Wakabayashi *et al.*, 2019), while, in cotton, a CYP722C homologue catalysed the conversion of CLA to 5-deoxystrigol (5DS) (Wakabayashi *et al.*, 2020) (Fig. 3). Mutant screening via forward genetics (i.e. by phenotype) in Arabidopsis has yielded most, but not all, genes in the SL pathway.

D27 was first identified in rice by forward genetics (Lin *et al.*, 2009). Based on knowledge of physiology and hormone interactions in the branching network (Hayward *et al.*, 2009; Beveridge and Kyozuka, 2010), co-expression analysis across three treatments and available Arabidopsis SL mutants yielded identification of LATERAL BRANCHING OXIDOREDUCTASE (LBO) (Brewer *et al.*, 2016) and D27 (Lin *et al.*, 2009; Alder *et al.*, 2012; Waters *et al.*, 2012a; Abuauf *et al.*, 2018) by reverse genetics (taking advantage of publicly available Arabidopsis stock mutants). The mutant for *LBO* has a weaker branching phenotype than other SL biosynthesis mutants, but the mutation is found to affect particular SL levels and branching phenotypes in diverse species (Walker *et al.*, 2019; Yoneyama and Brewer, 2021; Yang *et al.*, 2022). Based on biochemical and grafting studies, LBO acts downstream of MAX1 in Arabidopsis to produce a non-canonical SL (defined below). The *lbo* mutant was shown to accumulate MeCLA, a non-canonical SL which results from the methylation of CLA by the methyltransferase named CLAMT (Mashiguchi *et al.*, 2022). In contrast to CLA, MeCLA interacts with the Arabidopsis SL receptor (Abe *et al.*, 2014), suggesting that the methylation step is important for bioactivity (Fig.3).

**Fig. 4.**
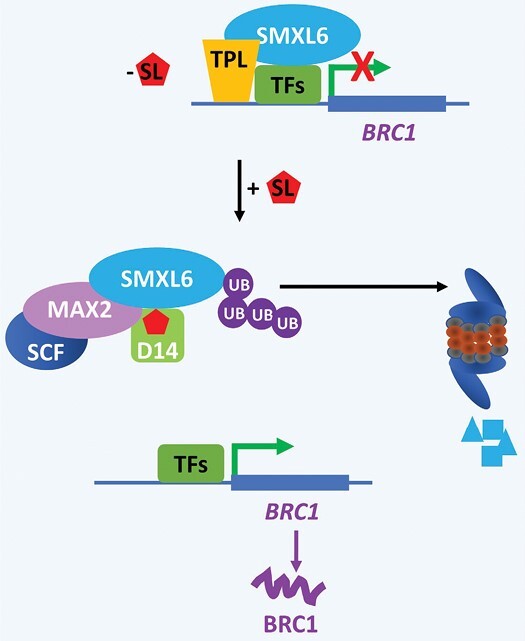
Simplified scheme of the SL signaling pathway. SL is perceived by D14, an alpha–beta hydrolase which hydrolyses SL into the ABC-ring and D-ring. The D-ring covalently binds to D14, and a complex with the MAX2 F-box and the SMXL6/7/8 proteins is formed, leading to ubiquitination-dependent degradation of SMXL/6/7/8. These proteins act as repressors of the SL signalling pathway by recruiting TPL co-repressors to repress the transcription of BRC1. In the presence of SL, degradation of the SMXL6/7/8 proteins releases the transcriptional activity of BRC1.

SLs are perceived by the α/β hydrolase D14 SL receptor, which forms an SL-dependent complex with the D3/MAX2 F-box protein (Hamiaux *et al.*, 2012; Zhao *et al.,* 2013; Yao *et al.*, 2016) (Fig. 4). This complex targets the rice DWARF53 (D53) or the Arabidopsis SUPPRESSOR OF MAX2 LIKE (SMXL6/7/8) for ubiquitination and degradation via the proteasome (Jiang *et al.*, 2013; Zhou *et al.*, 2013; Soundappan *et al.*, 2015; Liang *et al.*, 2016; Yao *et al.*, 2018b). The mechanism of SL perception is the subject of intense research and discussion as the SL receptor, which contains a catalytic triad conserved across species, acts both as a receptor and as an enzyme for SL hydrolysis (Nakamura *et al.*, 2013; de Saint Germain *et al.*, 2016; Snowden and Janssen, 2016; Yao *et al.*, 2016; Seto *et al.*, 2019; Tal *et al.*, 2022). Related key questions are whether the hydrolase activity is required for SL signalling and what is the exact order of events from SL perception to D53 or SMXL6/7/8 degradation (Tal *et al.*, 2022). The dominant high tillering dwarf mutant phenotype of d53 in rice, which is SL insensitive, indicated that D53 acts as a repressor of the SL signalling pathway to stimulate shoot branching (Jiang *et al.*, 2013; Zhou *et al.*, 2013). Consistent with this, the recessive *d53* mutation in the *d3* and *d14* signalling mutant backgrounds rescues the mutant phenotype of *d3* and *d14* to the level of the low tillering wild type (Fig. 4).

These genetic studies of SLs are important from an agricultural point of view as they provide genetic knowledge for consideration in crop improvement (Chesterfield *et al.*, 2020; Moreno *et al.*, 2021), improved understanding of SL perception in parasitic plants (Tsuchiya *et al.*, 2015, 2018; Lumba *et al.*, 2017; Uraguchi *et al.*, 2018; Y. Wang *et al.*, 2021), and genetic resources for researchers across diverse areas of plant development and symbioses that are affected by SLs (Xie *et al.*, 2010; Waters *et al.*, 2017; Bouwmeester *et al.*, 2019; Bürger and Chory, 2020; Yoneyama and Brewer, 2021; Alvi *et al.*, 2022).

Despite all this progress, the specific SL branching inhibitor(s) have not yet been unequivocally identified. To date, >30 natural SLs have been identified in root exudates of diverse species, and the number should increase when analysing other species (Yoneyama *et al.*, 2018b).

In 2023, the search for the specific SL branching inhibitor(s) is ongoing, along with the attribution of precise biological functions to different SLs in the many biological processes and phenotypes affected by SL (Yoneyama *et al.*, 2018b) (Fig. 3). SLs can be classified as canonical SLs and non-canonical SLs. The 23 already known canonical SLs show a tricyclic lactone ring system (ABC-ring) connected to a D-ring via an enol ether bridge, while non-canonical SLs have an unclosed BC-ring (Fig. 3). Recent studies have indicated that the SL-derived branching inhibitor would not be a canonical SL, but rather a non-canonical SL (Wakabayashi *et al.*, 2019; Ito *et al.*, 2022). Wakabayashi *et al.* (2019) characterized the CYP722 enzyme in cowpea (*Vigna unguiculata*) and tomato, which directly catalysed the formation of orobanchol from CLA. Surprisingly, the tomato knockout (KO) mutant for SlCYP722 did not display the bushy phenotype of the *Slccd8* mutant, despite its reduced levels of the canonical SLs, orobanchol and solanacol. Similarly, Ito *et al.* (2022) characterized the rice *Os900 max1* mutants as deficient in the rice canonical SLs, 4-DO and orobanchol. Surprisingly, the Os900-KO lines did not show the characteristic high tillering and dwarf phenotype of other SL-deficient mutants and even displayed fewer tillers than the wild type. These results indicate, together with other arguments (Yoneyama *et al.*, 2018b), that the SL branching inhibitor is a non-canonical SL.

Non-canonical SL (heliolactone, avenaol, and zealactone) are found in root exudates of sunflower (Ueno *et al.*, 2014), black oat (*Avena strigosa*) (Kim *et al.*, 2014), or maize (Charnikhova *et al.*, 2017), respectively, and were shown to act as major germination stimulants of parasitic plants. A non-canonical SL named bryosymbiol (BSB), has been recently discovered in the bryophyte *Marchantia paleacea* and has an essential function in the symbiosis with the AM fungi but has no obvious function on growth; BSB is also produced by several flowering plants (Kodama *et al.*, 2022).

In summary, mutants for enzymes in the core SL biosynthesis pathway (D27, CCD7, and CCD8) have a clear branching phenotype in multiple species, and branching can be inhibited in these mutants by SL, indicating that SLs are the precursors of the SL-related branching inhibitor. However, this SL-related branching inhibitor is still to be discovered and may be a non-canonical SL.

One significant difficulty in identifying or testing which SL(s) is the branching inhibitor is that identification of SLs in shoots is challenging. Other than the precursor, CL, the rare provided reports of SL in shoots highlight the very low levels (e.g. <10 pg g^–1^ FW of epi-5DS in the basal shoot of wild-type rice) (Umehara *et al.*, 2010). Furthermore, non-canonical SLs appear very diverse among different species (Yoneyama and Brewer, 2021). Another difficulty is that non-canonical SLs appear less stable than canonical SLs, which makes their isolation and their structural determination particularly difficult (Yoneyama *et al.*, 2018b).

It is likely that several hormone-like signals are yet to be discovered. For example, the KARRIKIN INSENSITIVE 2 (KAI2) protein, a close homologue of the D14 SL receptor, mediates the response to karrikins which are metabolites of the smoke of burned plant material and are involved in stimulating seed germination (Flematti *et al.*, 2004; Waters *et al.*, 2012b). It has been proposed that the function of KAI2 is to perceive an endogenous plant hormone, or a family of metabolites (Flematti *et al.*, 2013; Yao and Waters, 2020). Another well-established example of an unidentified hormone-like signal has been highlighted by studies with BYPASS1 (BPS1) which suppresses a root-to-shoot mobile signal, very probably carotenoid derived, that is required for shoot growth (van Norman and Sieburth, 2007; van Norman *et al.*, 2004, 2011). Given the unknown chemical identities of the SL branching hormone and other signals affecting plant development, it is important that we do not ignore them just for the sake of simplicity or a sense of a ‘complete story’.

## SL integration into the branching system

Grafting experiments demonstrated that SLs can be synthesized in both roots and shoots, and that they move in the plant primarily in a root to shoot direction (Foo *et al.*, 2001). The exact location of SL transport is still under debate, in particular whether SL can be found in the xylem sap. Until the SL branching inhibitor can be identified, SL transport through the xylem sap cannot be verified (Xie *et al.*, 2015; Mashiguchi *et al.*, 2021). The Petunia ATP-binding cassette (ABC) transporter PDR1 is located in the vasculature of shoots and in the node at the axil of leaves, possibly to transport SL into the bud (Kretzschmar *et al.*, 2012). As components of SL perception, D14 and MAX2 are expressed in the axillary buds (Stirnberg *et al.*, 2002; Arite *et al.*, 2009), and as the transcription factor BRANCHED 1 (BRC1) is mainly expressed in axillary buds of different species (Hubbard *et al.*, 2002; Takeda *et al.*, 2003; Aguilar-Martínez *et al.*, 2007; Braun *et al.*, 2012), it is very likely that SL acts locally in the bud for stimulating *BRC1* transcript levels (Martín-Fontecha *et al.*, 2018). Moreover, the direct application of SL onto the bud inhibits its outgrowth (Dun *et al.*, 2009; Boyer *et al.*, 2012). SL could also act at the node, or systemically in the main stem, for repressing auxin transport (Stirnberg *et al.*, 2007; Ongaro and Leyser, 2008). The developmental stage at which SL acts during bud outgrowth and sustained growth will be discussed in a later section.

SL mutants show increased branching, even though they maintain an actively growing shoot tip that is well capable of auxin transport (e.g. Beveridge *et al.*, 2000). Considering this, it is again worth commenting that increased branching is not the same as weak apical dominance because the branching control is not exerted solely from the shoot tip (Box 1). Nevertheless, both CK and SL can be thought of in some way as second messengers for auxin (Fig. 2A). Auxin is well established in its regulation of CK (as discussed above and by Nordström *et al.*, 2004; Tanaka *et al.*, 2006; El-Showk *et al.*, 2013). For SL, this is less clear. First, as mentioned above, the identity of the bioactive long-distance SL and shoot-acting SL is far from well established. Therefore, it is difficult to be sure that auxin regulates the SL branching inhibitor. Current evidence on auxin–SL interaction is from auxin regulation of SL pathway gene expression and genetic and physiological approaches that indicate that SL acts downstream of auxin (Beveridge, 2000; Foo *et al.*, 2005; Brewer *et al.*, 2009) (Fig. 2A). Auxin cannot inhibit branching in SL mutants, unless SL is supplied exogenously, through grafting, or if very high levels of auxin are supplied *in vitro* (Chatfield *et al.*, 2000; Young *et al.*, 2014; Bertheloot *et al.*, 2020). As discussed below, SL also acts independently of auxin regulation (Fig. 2).

It is clear that CK and SL actions as secondary messengers of auxin do not fully define these hormone roles. CKs and SLs are important branching regulators in their own right. SL branching mutants are not auxin depleted (Beveridge *et al.*, 1996, 1997b; Arite *et al.*, 2007), and changes in SL levels through environmental responses lead to concomitant changes in shoot branching that are largely auxin independent. For example, phosphate depletion typically causes enhanced SL levels and decreased branching—a relationship prevented in SL mutants that continue to branch under low phosphate (Umehara *et al.*, 2010). Similarly, changes in CK levels in the branching context may also occur independently of changes in auxin level or signalling (Foo *et al.*, 2007; Young *et al.*, 2014; Cao *et al.*, 2023). The role of sugars, nutrients, and their crosstalk with hormones is discussed again later.

## BRC1 as a central integrator

One of the key mysteries has been on the mechanisms of how CKs, auxin, and the SL branching inhibitor interact to regulate branching. A large part of the answer came following the identification of a highly conserved transcription factor: the TEOSINTE BRANCHED1, CYCLOIDEA, and PCF (TCP) transcription factor. TEOSINTE BRANCHED1 (TB1) in maize accounts for a considerable portion of the highly branched architecture of the maize progenitor compared with modern cultivated maize (Doebley *et al.*, 1997). The identification of Arabidopsis BRC1, an orthologue of TB1, demonstrated a strong degree of functional conservation of TB1/BRC1 across monocot and eudicot species. BRC1 was introduced as an integrator of signals in shoot branching (Aguilar-Martínez *et al.*, 2007; González-Grandío *et al.*, 2013; Rameau *et al.*, 2014; reviewed in Wang *et al.*, 2019) and is regulated by both CK and SL in diverse species without a requirement for protein synthesis (Braun *et al.*, 2012; Dun *et al.*, 2012) (Fig. 2). It took some years to demonstrate that TB1/BRC1/FC1 in rice was also transcriptionally regulated by both SL and CK (Xu *et al.*, 2015; Fang *et al.*, 2020) (Fig. 2), perhaps due to the comparatively difficult experimental challenge posed by the grass phenotype of rice where axillary buds are hidden inside a leaf sheath. The SMXL6/D53 SL repressor, one of the proteolytic targets of SL signalling, is able to bind to the *BRC1* promoter to repress its expression (L. Wang *et al.*, 2020). The role of CK and SL crosstalk is not specific to TB1/BRC1/FC1, as CKs also regulate D53/SMXL6 in rice and pea (Kerr *et al.*, 2021).

In Arabidopsis and pea, the *brc1* mutation is epistatic to the *smxl678* mutations (Seale *et al.*, 2017; L. Wang *et al.*, 2020; Kerr *et al.*, 2021); in particular, the null allele *brc1-6* was shown to completely suppress the shoot branching phenotypes of the triple mutant *smxl678*, suggesting that BRC1 is essential for the inhibition of shoot branching by SL (L. Wang *et al.*, 2020). In pea and rice, the *brc1* mutants were also shown to be less responsive than the wild type to SL (Aguilar-Martínez *et al.*, 2007; Braun *et al.*, 2012; Dun *et al.*, 2012; Guan *et al.*, 2012; Lu *et al.*, 2013) which would suggest that SL inhibition of shoot branching is largely dependent on BRC1. Nevertheless, in several species, when comparing the shoot branching of *brc1* mutants with that of SL-related mutants, a strong difference in intensity of branching and plant height can be observed, with the SL-related mutants being more dwarf and bushy than *brc1* mutants (Braun *et al.*, 2012; Bennett *et al.*, 2016). Moreover, partial inhibition of branching by a synthetic analogue of SL, GR24, can still be observed in *brc1* mutants (Seale *et al.*, 2017). These observations indicate that SLs regulate shoot branching via BRC1-dependent and BRC1-independent pathways. While further research should explore BRC1-independent sugar signalling more deeply, current evidence supports a role for SL-regulated auxin transport in non-transcriptional and BRC1-independent regulation of shoot branching (Seale *et al.*, 2017).

The molecular control of shoot branching by the MIR156–SPL (SQUAMOSA PROMOTER BINDING PROTEIN-LIKE) module has been particularly well characterized in rice (Jiao *et al.*, 2010; Miura *et al.*, 2010), Arabidopsis (Xie *et al.*, 2017), and more recently in liverwort (Streubel *et al.*, 2023). OsSPL14, also named Ideal Plant Architecture 1 (IPA1) in rice, and the two IPA1 homologues in Arabidopsis, SPL9 and SPL15, can directly bind to the promoter of TB1 and BRC1, respectively, to regulate shoot branching (Lu *et al.,* 2013; Song *et al.*, 2017; Xie *et al.*, 2020). IPA1 is considered as one of the new green revolution genes (Wang and Wang, 2017).

## Auxin transport

During the physiology era discussed above, auxin transport was proposed as an alternative or adjunct to the nutrient diversion theory for promoting bud release. The discovery of SL enabled a direct test of whether SL functions to inhibit branching through mediating auxin canalization (and the flow of auxin from axillary buds relative to the main stem). SL deficiency in most species is correlated with impaired auxin transport and impaired auxin-mediated PIN-FORMED (PIN) transmembrane protein localization (Bennett *et al.*, 2006; Prusinkiewicz *et al.*, 2009; Liang *et al.*, 2010; Balla *et al.*, 2011; Domagalska and Leyser, 2011) (Fig. 2). SL mediates auxin transport through auxin feedback on PIN polarity, which is required for auxin canalization (Zhang *et al.*, 2020). Auxin canalization is necessary for several SL-regulated processes, including vascularization. In addition to regulation by SL, CK stimulation of bud outgrowth is also associated with PIN regulation and enhanced auxin transport (Waldie and Leyser, 2018) (Fig. 2). Importantly, as mentioned above, much of the effect of SL on auxin transport may be independent of BRC1 (van Rongen *et al.*, 2019).

The extent to which auxin canalization is important for bud outgrowth in different systems may depend on factors such as the extent of bud vascular development already formed, the level of metabolic activity already underway in the bud, and what stage the bud has reached in the transition from bud release through to sustained bud outgrowth (Sachs and Thimann, 1967; Cline 1997; Fig. 2C). Some changes in auxin transport, though correlative, may not be causal of bud release. For example, in garden pea, which has well-established vasculature into buds and shows auxin canalization during bud growth (Balla *et al.*, 2011), there is no effect of bud-specific suppression of auxin transport on bud release (Brewer *et al.*, 2015; Chabikwa *et al.*, 2019). Despite this, auxin transport clearly has a strong effect on the sustained outgrowth phase of pea (Chabikwa *et al.*, 2019; Cao *et al.*, 2023) and Arabidopsis (Paterlini *et al.*, 2021) (Fig. 2D). Further investigation is required on the regulatory role of auxin transport during different phases of bud growth from bud release through to sustained growth.

## Sugar and nutrient signalling

Sugars and nutrients were a strong focus of research during the physiology era, but were considered somewhat less important during the genetics era. The question that remained unanswered throughout these eras was: are sugars and nutrients just required for manufacturing plant components during growth, or is a more sophisticated control mechanism at play? Similarly, what of the nutrient diversion theory regarding hormone-directed flow of inorganic nutrients and sugars—is it still valid today?

The timing of bud outgrowth after decapitation was a critical clue in narrowing down the role of sugars as opposed to depleted auxin as a trigger for bud release after decapitation. Whereas auxin depletion occurs at a rate of ~1 cm h^–1^ (or double this for the leading edge of depletion; Morris, 1977; Morris *et al.*, 2005; Renton *et al.*, 2012), sugars can move at 100 cm h^–1^ (Mason *et al.*, 2014). Sugar levels increase rapidly after decapitation in the node and bud at a long distance from the site of decapitation (Mason *et al.*, 2014; Fichtner *et al.*, 2017). Exogenous sugar supply in intact plants can also rapidly promote bud outgrowth, even in the presence of an actively growing shoot tip (Mason *et al.*, 2014; F. Wang *et al.*, 2020; Kebrom and Doust, 2022). By sensing sugar status, such as after decapitation, plants with a long distance between the shoot tip and axillary buds can trigger bud release prior to the longer term drop in auxin content (Mason *et al.*, 2014; Barbier *et al.*, 2015a, b; Fichtner *et al.*, 2017, 2021a, b; Bertheloot *et al.*, 2020; M. Wang *et al.*, 2021).

The mutant approach is not straightforward for the sugar pathways, as perturbing such a central pathway can obviously lead to plant-wide effects of growth and development, and hence was not, until recently, a viable approach for testing the role of sugars in shoot branching. Sugars are now widely recognized as signalling molecules and not only as a major source for carbon metabolism (Fichtner *et al.*, 2021a,b). With the ability to target expression with inducible systems or within particular tissues or to modify specific aspects of sugar signalling proteins, it is now more feasible to explore the molecular mechanisms of sugar signalling in plants. Several sugar signalling pathways have been associated with bud outgrowth. Trehalose 6-phosphate (Tre6P) is a low-abundance substance that reflects the sucrose status of the plant (Fichtner *et al.*, 2020) and, from some perspectives, may itself be considered a plant hormone (Paul *et al.*, 2018; Fichtner and Lunn, 2021; Fichtner *et al.*, 2021b). Arabidopsis plants engineered with altered levels of Tre6P show that Tre6P enhances shoot branching. Tre6P changes targeted to axillary buds with the BRC1 promoter have clear branching phenotypes that are not associated with highly pleiotropic phenotypes (Fichtner *et al.*, 2021a). In pea, the timing of enhanced levels of endogenous Tre6P after decapitation is associated with the onset of bud release (Fichtner *et al.*, 2017). As enhanced Tre6P levels cause additional branching in *brc1* mutants, it is likely that Tre6P acts at least partly independently of BRC1 (Fichtner *et al.*, 2021a).

Non-metabolizable sugars also provide a means of exploring sugar signalling without providing a carbon source for growth and are therefore a useful tool for demonstrating a role for sugar signalling (Rabot *et al.*, 2012; Barbier *et al.*, 2015b). For example, mannose can trigger the signalling component of HEXOKINASE1 (HXK1), an enzyme involved in glucose metabolism and sensing (Moore *et al.*, 2003), and promotes branching in rose and pea (Barbier *et al.*, 2021). Highlighting a signalling role for HXK1, mutants with completely disrupted HXK1 have a decreased branching phenotype which is largely reverted to wild type by transformation with a HXK1 bearing signalling activity but without catabolic activity (Barbier *et al.*, 2021).

Another line of evidence that sugars act as developmental regulators rather than simply as requirements for manufacturing growth is their influence on hormone signalling (Fig. 2). A comprehensive study in rose (Barbier *et al.*, 2015b) indicated considerable effects of sugars on the branching hormone network. This work showed that very high doses of exogenous auxin could over-ride the promoting effect of sugars and therefore partly explains why the early studies with exogenous auxin prompted exclusion of a role for sugars/sugar signalling in shoot branching (Cline, 1997; Morris *et al.*, 2005; Barbier *et al.*, 2015a; Bertheloot *et al.*, 2020). Genetic and molecular physiology studies demonstrate that part of the effect of sugars on bud outgrowth is mediated by the SL pathway in rose, pea, and rice, particularly through sugar modulation of SL signalling (Barbier *et al.*, 2015b, 2021; Bertheloot *et al.*, 2020; Patil *et al.*, 2022). The highly abundant metabolite, citrate, has recently been shown to reduce activity of the MAX2 SL signalling component, although it is not yet clear if citrate levels affect shoot branching (Tal *et al.*, 2022).

The overall supply of compounds to buds was proposed as important during the physiological era discussed above and needs to be reconsidered with respect to a wide range of mobile substances including sugars, but also mobile proteins such as the D14 receptor, the transport of which appears to be needed to fully inhibit tillering/branching in rice and pea (Kameoka *et al.*, 2016). Cell-specific genetic manipulation of phloem transport, fluorescent transport markers, and inducible systems are now able to be applied to address this question (Paterlini *et al.*, 2021). In contrast to perennial plants where callose deposition is often associated with bud endodormancy (including the apical bud; Wu *et al.*, 2018), axillary bud release in Arabidopsis was not significantly affected by increased callose deposition, and subsequent growth of Arabidopsis axillary buds was only somewhat suppressed (Paterlini *et al.*, 2021). This observation and other studies in grass crops led Kebrom and Doust (2022) to propose that perhaps apoplastic transport to dormant buds in annual plants may be more important than symplastic transport.

Carbon starvation (C-starvation) has also been suggested as a mechanism for inducing bud dormancy (Tarancón *et al.*, 2017; Martín-Fontecha *et al.*, 2018). Meta-analyses of three transcriptomic datasets in Arabidopsis comparing active and dormant buds either in decapitation or in light treatment experiments (Tatematsu *et al.*, 2005; González-Grandío *et al.*, 2013; Reddy *et al.*, 2013) showed transcriptional responses typical of a C-starvation response. Accordingly, dormant buds were associated with the up-regulation of typical dark-induced, sugar-repressed genes, or genes induced by the protein kinase sucrose non-fermenting 1 (SNF1)-related protein kinase 1 (SnRK1), which is a key metabolite sensor that triggers energy-saving programmes in response to stress and low energy signalling (Baena-González *et al.*, 2007; Nukarinen *et al.* 2016; Baena-González and Hanson 2017). This kinase is inactivated by Tre6P (Zhang *et al.*, 2009; Zhai *et al.*, 2018) and activated by ABA signalling (Rodrigues *et al.*, 2013; Belda-Palazón *et al.*, 2020, 2022). The same gene regulatory networks related to C-starvation were also identified in buds of poplar and grapevine during the growth to dormancy transition. Therefore, the authors proposed that bud dormancy (eco-, endo-, and paradormancy; Box 1) could be a consequence of this carbon starvation syndrome (Tarancón *et al.*, 2017; Martín-Fontecha *et al.*, 2018).

Regarding inorganic nutrients, there is clearly a role in shoot branching. In several species, SL levels in roots and root exudates are highly regulated by phosphate (P) and nitrogen (N) (Yoneyama *et al.*, 2012; Andreo-Jimenez *et al.*, 2015; Mashiguchi *et al.*, 2021). Under low nutrient conditions, which favour AM symbiosis, SL levels are greatly enhanced and are associated with decreased branching. In SL synthesis and signalling mutants, this relationship between low nutrients and branching inhibition is severely impaired, indicating that a component of the P and N nutrient effect on branching is via SL signalling and not simply due to the requirement for P and N in manufacturing plant growth (Umehara *et al.*, 2010; Yoneyama *et al.*, 2012; de Jong *et al.*, 2014; Sun *et al.*, 2014). A somewhat similar argument can be made for the nutrient regulation of CK (Sakakibara *et al.*, 2006; Ramireddy *et al.*, 2018). Several recent studies in rice decipher the molecular mechanisms by which nutrient signals (nitrogen in particular) and hormonal signals (GA, brassinosteroid, and SL) are integrated for regulation of tillering (Guo *et al.*, 2013; Huang *et al.*, 2019; Wu *et al.*, 2020; Liu *et al.*, 2021).

The realization that changes in sugars and sugar signalling occur prior to the local changes in auxin content in the stem led to several implications and predictions, including for the role and timing of auxin-regulated changes in SL and CK content (Fig. 1). Recent work in pea has established that the rapid sugar changes after shoot tip removal (Mason *et al.*, 2014) probably regulate CK levels in buds (Cao *et al.*, 2023) (Fig. 2). This sugar induction of CK levels (Salam *et al.*, 2021) can occur prior to changes in auxin content in the stem (Cao *et al.*, 2023). Quite strikingly, it is also clear that both sugars and CKs attenuate the effect of SLs (Bertheloot *et al.*, 2020; Barbier *et al.*, 2021; Kerr *et al.*, 2021; Salam *et al.*, 2021). Accordingly, SLs in pea are not effective at inhibiting bud release immediately after decapitation (Cao *et al.*, 2023) which coincides with the period of enhanced sugar and Tre6P levels (Fichtner *et al.*, 2017).

Rapid increases in auxin levels in buds as reported during the physiology era (e.g. Gocal *et al.*, 1991) are now thought to be at least partly caused by the rapid increase in sucrose levels after decapitation (Mason *et al.*, 2014; Barbier *et al.*, 2015b; Ljung *et al.*, 2015; Cao *et al.*, 2023). Given the established positive effect of auxin on GA content, which leads to elongation growth, additional clear evidence for the role of GA (Ross *et al.*, 2000, 2003; O’Neill and Ross, 2002) has again resurfaced due to evidence of tightly correlated levels of IAA and GA in axillary buds of pea during bud outgrowth (Cao *et al.*, 2023). GA is ineffective as a growth stimulant in dormant buds but is highly effective at inducing sustained growth after bud release (Cao *et al.*, 2023, and references within) (Fig. 2D). Interestingly, at least in roots of rice, GA suppresses SL levels (Ito *et al.*, 2017). If this was the case during sustained growth in buds, it would reinforce the activation of bud growth. Similarly, with respect to having a role in sustained growth, auxin transport and auxin signalling inhibitors are not able to inhibit bud release (Brewer *et al.*, 2015; Chabikwa *et al.*, 2019) but do inhibit sustained growth (Fig. 2D). Applied exogenously to buds, GA can overcome auxin inhibition to promote sustained growth, indicating that part of the mechanism of auxin regulation of sustained growth is via GA. This model is easy to test in pea due to its architecture of exposed buds and long internodes. Although the model can also apply to Arabidopsis and rice (Fig. 2), it is more challenging to test for these species. The association between bud elongation in Arabidopsis and enhanced GA content at flowering and bolting makes it difficult to identify causal effects. In rice and many other species including pea, dwarfism is associated with enhanced tillering or branching, but again it is challenging to tease apart the effect of dwarfism on sugar availability to axillary buds with potentially direct effects of GA on sustained growth.

## What about ABA?

ABA has long been in the picture for being involved in shoot branching (Fig. 1). In perennial plants, ABA has a key role in the photoperiodic control of bud dormancy and, in hybrid aspen, ABA was shown to block symplastic communication during endodormancy (Tylewicz *et al.*, 2018). During water deficiency, ABA accumulates in the bud and potentially inhibits bud outgrowth (Jones *et al.*, 1976; Rinne *et al.*, 1994; Demotes-Mainard *et al.*, 2013). For studies of herbaceous annuals during the physiology era, ABA was proposed (Tucker, 1977) and then re-evaluated (Cline and Oh, 2006) as a candidate for the secondary messenger and was more recently suggested to act downstream of BRC1 (González-Grandío *et al.*, 2013, 2017). The biological role of ABA in inhibition of axillary buds is still not clear. Genetic studies indicated that ABA was not a good candidate for the secondary messenger because auxin inhibition of bud outgrowth was not affected in ABA-insensitive mutants (Chatfield *et al.*, 2000) and ABA mutants do not collectively exhibit altered branching phenotypes. However, several mutants with increased branching do have reduced ABA levels or signalling (Yao and Finlayson, 2015). Various transcriptomic studies have identified ABA-related genes associated with suppressed buds (González-Grandío *et al.*, 2013; Reddy *et al.*, 2013; Luo *et al.*, 2019), and a large proportion of these ABA-related transcriptional changes are BRC1 dependent (González-Grandío *et al.*, 2013, 2017) (Fig. 2). Consistent with this, branching in the wild type and SL mutants can be suppressed by elevated levels of ABA (Luo *et al.*, 2019).

The extent to which ABA is critical for bud outgrowth is still an open question requiring further investigation and may be related to cellular stress management. As discussed above, dormant buds in annual plants remain metabolically active and are poised to enable rapid outgrowth. How then do buds retain this ability whilst not growing? It probably involves stress management and autophagy and the C-starvation syndrome, processes of which include a central role for ABA (Liao and Bassham, 2020). It is therefore possible that enhanced ABA levels and signalling in dormant buds (Fig. 2B) may be due to these other reasons associated with dormancy and quiescence rather than ABA acting to specifically inhibit bud growth. Reminiscent of the scenario discussed above of high supplied auxin levels masking the role of sucrose, we are yet to discover whether endogenous ABA levels are causal of bud inhibition, rather than, for example, being required for processes that sustain dormant buds during their inhibition.

## Role of light in shoot branching

BRC1 is also a key integrator of light quality, in particular the response to plant density and to changes to the ratio of red to far-red (R:FR) wavelengths of light (Finlayson *et al.*, 2010; González-Grandío *et al.*, 2013). Reduced shoot branching is part of the shade avoidance response which commonly also includes increased plant height and early flowering time (Casal, 2012; Fichtner *et al.*, 2022). In sorghum and Arabidopsis, it was shown that high planting density and a low R:FR ratio results in high TB1/BRC1 expression and reduced shoot branching/tillering (Kebrom *et al.*, 2006; Aguilar-Martínez *et al.*, 2007; Finlayson *et al.*, 2010; González-Grandío *et al.*, 2013). Xie *et al.* (2020) provide evidence that phytochrome A-mediated light signalling, acting via FHY3/FAR1 transcription factors in Arabidopsis, regulates BRC1 via the previously mentioned conserved components SPL9/SPL15 and SMXL6/SMXL7/SMXL8. This light signalling also affects ABA levels (Holalu and Finlayson, 2017), and ABA-deficient mutants display a reduced branching inhibition in response to low R:FR light ratios (Reddy *et al.*, 2013). A significant role for BRC1 that up-regulates ABA levels and signalling in buds was proposed based on ABA quantification and the expression of ABA-related or responsive genes in *brc1* mutants and inducible BRC1 lines (González-Grandío *et al.*, 2013, 2017; González-Grandío and Cubas, 2014). Interestingly, in addition to the well-established effects of light quality on shoot architecture, two recent studies (Wheeldon *et al.*, 2022; Yoneyama *et al.*, 2022) have proposed that plants can also detect neighbours through sensing SL levels in the rhizosphere.

## Future directions and perspectives

Time-lapse video of growing buds indicates that growth is widespread across external bud surfaces, including the basal internode and the outer leaves that encapsulate the rest of the bud (e.g. Mason *et al.*, 2014). However, very little attention has been given to the sequence and timing of anatomical events during bud outgrowth, including cell division and elongation. There is also no elucidation as to which anatomical parts are essential for the bud’s responses to endogenous or exogenous signals.

It is clear that the core components of the branching network are conserved across the model herbaceous species pea, petunia, Arabidopsis, and rice. As described above, some details are conserved but are more challenging to experimentally verify due to, for example, bud size, transformation efficiency, or growth habit. Some mechanistic details, such as the SL transporter PDR1 in petunia, may be more species specific (Kretzschmar *et al.*, 2012; Borghi *et al.*, 2016). Perhaps even more importantly, evolution may have selected many overlapping control mechanisms. Given such complex systems, we should not always question this ‘or’ that but, instead, question the relative contribution of this ‘and’ that. The latter framing of the question requires a more complex but potentially more rewarding research approach.

As mentioned above, the SL-derived branching inhibitor has yet to be identified chemically. Feedback regulation of SL biosynthesis gene expression across multiple genes has not been well understood. It probably includes another unidentified long-distance signal as well as a branch-derived signal which may relate to correlative inhibition and/or sugars (Dun *et al.*, 2009). The role of auxin transport in maintaining sustained growth needs further inquiry. It may relate well to the original auxin work regarding competition between shoots (correlative inhibition), rather than apical dominance (Cline, 1997).

An intriguing question concerns the role of CK in the root xylem sap in shoot branching, which is substantially regulated by a shoot to root feedback signal in the pea *rms* mutants and in the Arabidopsis *max* mutants, and is regulated differently from overall shoot or root CK levels (Beveridge *et al.*, 1997a; Foo *et al.*, 2007; Young *et al.*, 2014). The grafting experiments of wild-type scions on CK-overproducing transgenic ISOPENTENYL TRANSFERASE (IPT) rootstocks of tobacco showed that branching of wild-type shoots was not observed despite high root-derived CK levels and suggested that CK synthesized in shoots is essential for bud outgrowth (Faiss *et al.*, 1997). In contrast, Dun *et al.*, 2009, 2012 show that xylem-derived CKs may have a role.

The *RMS2* (*PsAFB5*) gene in pea that affects long-distance feedback regulation of SLs and CKs is a member of the auxin receptor family (Ligerot *et al.*, 2017). Studies with *RMS2* and SL mutants in pea and Arabidopsis have been useful in demonstrating that SLs suppress auxin biosynthesis (Ligerot *et al.*, 2017) and transport (Bennett *et al.*, 2006; Prusinkiewicz *et al.*, 2009). Intriguingly, the Arabidopsis *afb4/5* mutant and the pea *rms2* mutant display a specific resistance to the synthetic herbicide picloram. Therefore, it is possible that the auxinic compound perceived by AFB5 has yet to be discovered. Finally, perceived reduced SL levels in shoots led to reduced xylem sap CK levels in rootstocks (Beveridge *et al.*, 1997a; Foo *et al.*, 2007; Sakakibara, 2021), demonstrating yet another unidentified long-distance signal. There are many transcription factors and other genes not reviewed here that directly or indirectly affect shoot branching and yet have not been placed into the networks described here (e.g. Wu *et al.*, 2020; Li *et al.*, 2021; Xia *et al.*, 2021; Zhang *et al.*, 2022; Hellens *et al.*, 2023).

Given that the overall interactions among auxin, SL, and CKs (Fig. 2) are conserved for regulating branching in diverse plants, this physiological network structure must have a very strong fitness benefit with respect to plant architecture under natural conditions. Although differences exist among species, the conserved structure of the core of the branching network is of considerable value in understanding the importance of different aspects of branching control and in implementing agricultural solutions relating to plant architecture (Powell *et al.*, 2022). It is hoped that through modelling this network and integrating it with crop modelling platforms (e.g. APSIM, Holzworth *et al.*, 2018), we can simplify the complex trait/gene×environment×management (G×E×M) interactions that impede genomic prediction and the breeding process (Passioura, 2020; Cooper and Messina, 2023).

Of obvious note to any observer is that the rate and complexity of scientific discovery are increasing (Fig. 1). Emerging early career researchers are faced at the outset of their careers with a huge amount of data and knowledge that one can only assume is daunting at best. Even with the benefits of machine learning and modern technologies, science is at risk of ignoring so much profound knowledge that has been attained over the scale of a century and which continues to be produced (Fig. 1). How do we escalate the re-emergence of theories largely ignored in the past (e.g. nutrient diversion theory) or revive forgotten information without simply re-discovering it? Language and accessibility remain a huge barrier for many scientists in our global community, and hence attempts to catalogue information publicly and independently of language, gender, and culture should be encouraged, particularly in view of the current rate of discovery (Amano *et al.*, 2021). Curated knowledge graphs can visualize and analyse results (ideas) as a network (Ji *et al.*, 2022). There are a number of products emerging that will enhance the capacity of researchers and others to access and connect information such as https://knetminer.com/. Perhaps the next *JXB* Centenary review will involve such a cloud-type database of knowledge that describes the whole picture of our knowledge as a directed network, perhaps presented like a gene interaction correlative network. This type of approach could be used to link the strength of evidence to discoveries without unnecessary bias towards recent knowledge, researcher perspective, language, or accessibility.
